# Supplementary material for: Comparison of Physicians’ Attitudes and Practice Regarding Vaccination during Pregnancy in Turkey
Source: Vaccines (Basel). 2024 Jul 18;12(7):798. doi: 10.3390/vaccines12070798 (PMC11281721; doi:10.3390/vaccines12070798)
Supplement: Supplementary file 1 [file vaccines-12-00798-s001.zip › vaccines-3025979-supplementary.pdf]

THE SURVEY ABOUT ”PHYSICIANS' APPROACHES TO IMMUNIZATION DURING PREGNANCY"

Question 1: Are you actively working as a Pediatrician / Obstetrician and Gynecologist / Family Physician?

a) Yes
b) Please leave the survey

Question 2: Do you recommend vaccination during pregnancy?

Vaccines	Recommended routinely	Recommended in case of a risk	Not recommended
Influenza Vaccine			
Td (Tetanus and Diphtheria) Vaccine			
Tdap (Tetanus, Diphtheria, and Pertussis) Vaccine			

Question 3: Do you think that vaccination during pregnancy is an effective method to protect mother and newborn against infectious diseases?

Vaccines	1: not effective at all	2	3: uncertain	4	5: very effective
Influenza Vaccine					
Td (Tetanus and Diphtheria) Vaccine					
Tdap (Tetanus, Diphtheria, and Pertussis) Vaccine					

Question 4: Do you think vaccination during pregnancy is safe for mother and newborn?

Vaccines	1: not safe at all	2	3: uncertain	4	5: very safe
Influenza Vaccine					
Td (Tetanus and Diphtheria) Vaccine					
Tdap (Tetanus, Diphtheria, and Pertussis) Vaccine					

Question 5: Do you think vaccination during pregnancy is safe for mother and newborn?

	1: not effective at all	2	3: uncertain	4	5: very effective
Pregnant women generally think that vaccines are safe.					
Pregnant women think that there is not enough medical data to receive the vaccine.					
Pregnant women are afraid to receive any vaccine, even if there is sufficient medical data.					
Pregnant women think that the vaccine is necessary to protect their babies and themselves.					
Pregnant women think protecting their babies with postnatal vaccination is sufficient.					
Explaining the disease risks and benefits of vaccination to pregnant women increases the acceptance rate of the vaccine.					

Question 6: Do you have any knowledge of the disease burden of whooping cough?

a) Yes
b) Uncertain
c) No

Question 7: If you were pregnant, would the vaccination recommendation of your obstetrician be sufficient for your decision to get vaccinated?

a) My doctor's recommendation would have been sufficient
b) I would decide after searching the issue
c) I wouldn't get vaccinated

Question 8: If you were pregnant, would the vaccination recommendation of your pediatrician who followed up with your other child be sufficient for your decision to get vaccinated?

- **a)** My doctor's recommendation would have been sufficient
- **b)** I would decide after searching the issue
- **c)** I wouldn't get vaccinated

Question 9: Do you need detailed information about vaccination during pregnancy?

- **a)** Yes
- **b)** No
